# Real-World fNIRS Brain Activity Measurements during Ashtanga Vinyasa Yoga

**DOI:** 10.3390/brainsci11060742

**Published:** 2021-06-03

**Authors:** Henrikke Dybvik, Martin Steinert

**Affiliations:** TrollLABS, Department of Mechanical and Industrial Engineering, Norwegian University of Science and Technology (NTNU), 7491 Trondheim, Norway; martin.steinert@ntnu.no

**Keywords:** fNIRS, cognitive load, human cognition, real-world, in situ, ecological validity, ashtanga, yoga

## Abstract

Functional near-infrared spectroscopy (fNIRS) is often praised for its portability and robustness towards motion artifacts. While an increasing body of fNIRS research in real-world environments is emerging, most fNIRS studies are still conducted in laboratories, and do not incorporate larger movements performed by participants. This study extends fNIRS applications in real-world environments by conducting a single-subject observational study of a yoga practice with considerable movement (Ashtanga Vinyasa Yoga) in a participant’s natural environment (their apartment). The results show differences in cognitive load (prefrontal cortex activation) when comparing technically complex postures to relatively simple ones, but also some contrasts with surprisingly little difference. This study explores the boundaries of real-world cognitive load measurements, and contributes to the empirical knowledge base of using fNIRS in realistic settings. To the best of our knowledge, this is the first demonstration of fNIRS brain imaging recorded during any moving yoga practice. Future work with fNIRS should take advantage of this by accomplishing studies with considerable real-world movement.

## 1. Introduction

Functional near-infrared spectroscopy (fNIRS) is a non-invasive, lightweight, and portable neuroimaging technique which measures cortical brain activity [1,2]. fNIRS uses optical fibers to emit near-infrared light into a region of the brain, and detect changes in blood flow oxygenation (oxygenated (ΔHbO)) and deoxygenated hemoglobin (ΔHbR), caused by neural activation [1]. The light of different wavelengths in the near-infrared (NIR) spectrum penetrates the scalp and travels through different layers of the head, before reaching neuronal tissue. Inside the tissue, NIR light is absorbed differently in hemoglobin depending on the oxygen saturation state. Non-absorbed light scatter components are detected, and ΔHbO and ΔHbR are calculated by the modified Beer-Lambert Law. Neural activity induces changes in local hemodynamics, causing an increase in HbO concentration in the activated region, and a decreased concentration of HbR [1,2,3] (although this is not always the case [4]). This is used to measure cognitive states and cognitive load [5,6,7,8].

For cerebral hemodynamics, fNIRS can act as a surrogate for functional magnetic resonance imaging (fMRI) [9,10,11]. FNIRS is not limited to the restrictive fMRI environment, and since it is relatively robust against motion artifacts, the technique allows for freely moving participants in contexts with high ecological validity and in the real world (or in situ) [1,2,12]. Examples of such studies include, but are not limited to, outdoor activities, such as riding a bike [13] and walking [14]; farm workers at individual farm locations [15]; driving a car on an expressway [16]; setting a table [17]; radiologists interpreting MRI and CT images [18]; exposure therapy of arachnophobia [19]; performing penalty kicks in soccer [20]; playing table tennis, playing the piano, and human interaction during a violin duo [12]. Such studies are important since they may help us understand how the brain functions in real-life situations. They may also allow us to detect brain activity that can only be detected during movement. For example, one study revealed that the cortical activation from conducting an everyday task was not detected during an imitation of the same task [21]. To investigate and understand brain activity during *any* activity or task, it is, therefore, best to measure it directly in the environment where it naturally occurs (in situ). In situ studies may, for example, be beneficial when analyzing sports or strenuous exercise, social interaction in natural environments [13], operators at work (air traffic controllers [22], captains [23], and drivers [24,25]), and walks in nature. Moreover, when studying populations who may not be able to come to the lab (e.g., severe Alzheimer’s patients [26]), or when coming to a lab would be counterproductive to the topic of interest (e.g., during physical therapy and rehabilitation [27,28]), it may be necessary to conduct the study in the participant’s own environment.

FNIRS is often praised for its portability and robustness towards motion artifacts. However, most fNIRS studies are still conducted in laboratories today. An increasing body of research uses fNIRS in real-world environments at moderate levels of motion; indeed, several of the studies mentioned above include moderate levels of motion, e.g., [12], but none incorporate considerable or vigorous movements. We believe that fNIRS can be applied in real situations to a greater extent than it is currently. There is a need for in situ fNIRS studies with considerable movement.

Thus, the aim of this study was to extend fNIRS applications in real-world environments by recording fNIRS during a moving yoga practice in a participant’s natural environment. Ashtanga Vinyasa Yoga is a practice with considerable movement and complex postures, which may have some effect on cognitive functions. Therefore, we explored changes in brain activity (prefrontal cortex activation) within postures in the Ashtanga primary series. The research objectives were as follows: (1) Test the feasibility of fNIRS recordings during a yoga practice with considerable movement. (2) Test if different yoga postures have different cognitive loads. To this end, a single-subject observational study was adopted, in which one participant practiced Ashtanga with a wearable fNIRS in their own apartment for a total of seven times. The results show differences in cognitive load when comparing technically complex postures to relatively simple ones, but also some contrasts with little difference, although a greater difference was hypothesized. The fNIRS measurements taken during Ashtanga Vinyasa Yoga deepen our understanding of the effect of yoga postures and thus contributes to the scientific foundation of yoga. This study explores the boundaries of cognitive load measurements in the real-world, and contributes to the empirical knowledge base of using fNIRS in realistic settings.

## 2. Background

### 2.1. Yoga

The term “Yoga” denotes a group of physical, mental, and spiritual practices originating in ancient India [29,30,31]. Today, modern schools of yoga and thus styles of yoga each have a distinct relative content of ethics (*yama* and *niyama*), physical postures and exercises (*asanas*), breathing techniques (*pranayama*), and meditation practices, which aims to cultivate awareness; unite the mind, body, and spirit, alleviating suffering; and ultimately obtain profound states of consciousness [29,30,31,32]. Meditation practices include sensory withdrawal (*pratyahara*), concentration (*dharana*), meditation (*dhyana*), and a deep level of concentration (or absorption) described as self-transcendence (*samadhi*) [30].

### 2.2. Ashtanga Vinyasa Yoga

Ashtanga Vinyasa Yoga (Ashtanga for short) is a popular and physically demanding yoga style [33,34,35]. It is known for its vigorous flow, which may be why some adaptations of the practice are known as power yoga [36]. In Ashtanga, physical postures (*asanas*) are linked by flowing movements (*vinyasas*) and synchronous breathing techniques (*pranayama*) [35,36,37]. An Ashtanga session begins with sun salutations as a warmup, followed by a predefined sequence of postures, and a closing sequence. A total of six series exists, each with different sequences. The primary series is often called yoga therapy or yoga for health. It focuses on health healing effects, the release of trapped emotions, and raising and overcoming emotional and other unhealthy habitual patterns [37,38]. Ashtanga focuses on the coordination of posture, breath, and gaze [34]. These components form the *Tristana*, which is unique to Ashtanga [34]. A strong focus on physical embodiment is necessary since the postures are technically complex, and each movement is coordinated with an inhale or exhale, while the postures are held for five breaths [34,39]. The breathing technique is called *Ujjayi Breathing*, or victorious breath [39]. Each posture and movement has a specific gaze point intended to reduce external distractions and induce concentration (e.g., navel-gazing or *omphaloskepsis*, which is defined as the “contemplation of one’s navel as an aid to meditation” [40]) [34]. The repetitive practice is intended to move practitioners towards a control of mental activity that enables true self-realization [37,38]. Due to this highly focused attention during bodily movements, yoga is often called “meditation in motion” [30]. The rigid adherence to a standardized and documented posture series makes Ashtanga a strong candidate for scientific study [35].

### 2.3. Existing Research on Yoga

An increasing body of research shows positive effects from yoga practices and interventions on physical and psychological health [29,30]. Symptoms of depression, PTSD, epilepsy, ADHD, stress, and anxiety have been alleviated with yoga-based therapies. A reduction in stress and anxiety symptoms is also found in healthy individuals [30,41]. Yoga practitioners report increased psychological wellbeing, life satisfaction, happiness, motivation, and relaxation [30,36,42]. Reduced levels of galvanic skin response and blood lactate have been measured [41], along with improvements in physical fitness, and reduced sympathetic nervous system activity [33,39]. Some can also reduce their heart rate voluntarily without external cues [32]. Ashtanga practitioners specifically show improvements in muscular strength, endurance, flexibility, health perception, diastolic blood pressure, perceived stress [39], cardiac and respiratory fitness [33], and self-transcendence [43]. The results of Ashtanga intervention studies show significant improvements in psychological wellbeing, self-esteem, assertiveness, attention to one’s needs, and capacity to connect [34,44].

Several yoga techniques claim to enhance cognitive and executive functions, such as attention/awareness, concentration, emotion regulation, and cognitive control. Studies of such found greater gray matter volume, increased functional connectivity, improved cognitive performance, the strengthening of interoceptive and executive/control networks for yoga practitioners, and decreased glucose metabolism (which is linked to the improved regulation of negative emotions) [45]. Both elderly and adolescent practitioners have significantly improved cognitive performance [46,47]. A functional near-infrared spectroscopy (fNIRS) study found increased blood flow (measured by HbO concentration) to the dorsolateral prefrontal cortex during a yoga breathing technique [48] and increased bilateral blood flow to the prefrontal cortex in yoga practitioners compared to non-practitioners during sustained attention [49]. Electroencephalograms (EEGs) have also been conducted during various yoga practices with little movement [50]. In [30], the findings are consistent with the notion that yoga can improve cognitive regulation, to the point of offsetting the age-related decline in fluid intelligence in practitioners. They further suggest that this may be explained by the increased availability of neural resources, and postulate that neuronal interactions occurring during yoga practice include the cortical regions (i.e., the dorsolateral prefrontal cortex (DLPFC), anterior cingulate cortex (ACC), and orbitofrontal cortex) [30].

### 2.4. The Prefrontal Cortex

Executive and cognitive functions, such as attention/awareness, working memory, cognitive flexibility, and cognitive control and planning, are performed by the prefrontal cortex (PFC) [51,52,53,54]. The PFC synthesizes diverse information related to a given goal; it is responsible for planning and selecting complex cognitive behavior; and it is crucial for higher order processing [52]. Further studies relate cognitive control to activity in dorsolateral PFC (DLPFC) [55]. DLPFC plays an important role in the anticipatory organization of action and effortful tasks [56,57]. The mid-dorsolateral PFC (mDLPFC) aids in planning action sequences (organization external/internal action), i.e., mental conception and evaluation of behavioral sequences and associated outcomes before execution [56]. The frontopolar cortex (FPC) is involved in mind wandering, planning, abstract reasoning, multitasking, and cognitive branching, which require switching away from an ongoing behavioral option, considering multiple behavioral options, and/or exploring new ones [58]. Thus, the FPC is suggested to make a crucial contribution to the exploration and rapid acquisition of novel behavioral options [59]. The medial FPC governs undirected exploration, i.e., monitoring the current goal for possibly redistributing cognitive resources to other potential goals. The lateral (right/left) FPC cortex governs directed exploration, i.e., monitoring a few alternative tasks/goals for possibly re-engaging one as a replacement of the current task/goal [58].

## 3. Materials and Methods

Given that there are cognitive benefits of yoga practices, and that executive and cognitive functions are governed by the PFC, our aim was to determine whether we could measure any of them. Specifically, we investigated whether there were differences in cognitive load and cognitive state within postures in the Ashtanga primary series. The features of Ashtanga makes its practice a suitable context for demonstrating that brain activity can be measured during vigorous movement. To our knowledge, while intervention and laboratory studies of various yoga practices have been conducted, there is no study that incorporates neuroscientific measurements during a moving yoga session, nor in a real-world setting. We thus believe that this study is the first to record fNIRS during a moving yoga session.

### 3.1. Single-Subject Observational Study

A single-subject real-world (in situ) study of the half primary series in Ashtanga was conducted in the participant’s own living room. The participant was fitted with an fNIRS sensor cap aided by another person. The signal quality check, and the start and stop of data collection, was performed by the participant.

A video of the full primary series performed and led by Ty Landrum was used for instructional purposes [60]. In this video, the yogi practices the sequence and gives voice-over instructions with postures and queues. The yogi also performs the opening and closing mantra (or chant). The participant listened to the chants in Mountain Pose with their hands in prayer position. This video was used in all sessions. The practice was adapted to accommodate the head-mounted sensors, i.e., the participant refrained from postures requiring the head to be placed on the ground. Table A1 in Appendix A includes a list of the postures that were a part of the practice, and if they were adapted or not conducted. The postures were held for five long breaths. Repeated measures over time were made corresponding to the repeated postures over several full practices, as explained in the introduction.

### 3.2. Participant

The participant was 26–27 years old, female, right-handed, had corrected-to-normal vison, and had a good general physical fitness level. The participant had 3 months of practicing the primary series in Ashtanga once a week and was, therefore, considered a novice in Ashtanga. She had two years of experience practicing other yoga types at the time of recording. The participant was obtained by convenience sampling and had worn fNIRS ahead of this study.

### 3.3. Data Collection

**Figure 1:**
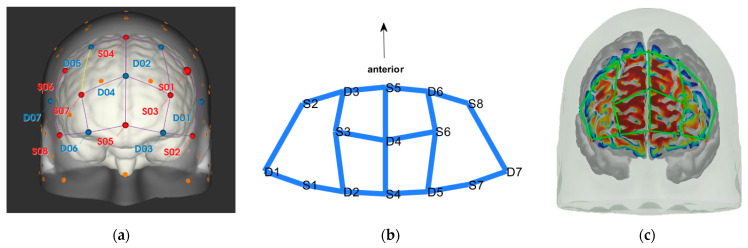
(**a**) The montage rendered onto the ICBM 152 Nonlinear atlases version 2009 [63,64], created with NIRSite 2020.7 (NIRx Medical Technologies). (**b**) The channels from above in an anterior orientation, created with NIRS toolbox [65]. (**c**) The sensitivity profile of the probe, created with AtlasViewer [62].

fNIRS data were sampled at 7.81 Hz by NIRSport (NIRx Medical Technologies, Berlin, Germany) with 8 sources and 8 detectors at two wavelengths (760 and 850 nm). Optodes were placed on the PFC, per montage by NIRx, as illustrated in Figure 1. The sources (denoted Sx) were placed as follows: S1: F3; S2: AF7; S3: AF3; S4: Fz; S5: Fpz; S6: AF4; S7: F4; S8: AF8. The detectors (denoted Dx) were placed as follows: D1: F5; D2 F1; D3 Fp1; D4 AFz; D5 F2; D6: Fp2; D7: F6. We used an EASYCAP AC-128-X1-C-58 (EASYCAP GmbH, Herrsching, Germany) with a 128-channel layout following the 10–5 system [61]. This montage covers the anterior frontal lobe, more specifically anterior regions of the right, left, and mid-dorsolateral PFC (l/r/mDLPFC), and right, left, and medial FPC (r/l/mFPC). A sensitivity profile of the montage/probe was generated with AtlasViewer [62] and is illustrated in Figure 1c.

In the bilateral PFC, HbO activation increases linearly with increasing cognitive load. In the PFC, an increase in functional connectivity between hemispheres and across hemispheres is associated with increasing cognitive load. Moreover, functional connectivity is different for different cognitive states [51].

NIRSport was connected via cables to a Dell Latitude 7490 laptop (with Microsoft Windows 10 Education, Intel(R) Core(TM) i7-8650U CPU @ 1.90 GHz 2.11 GHz processor, 32.0 GB RAM installed, 64-bit operating system, x64-based processor, and a 500 GB SSD harddrive). Nirstar 15.2 Acquisition Software (NIRx Medical Technologies) [66] was used to forward a continuous data stream through LabStreamingLayer [67] to iMotions 8.1 [68], which synchronized physiology data and video recordings. Two video recordings were made with a laptop-integrated web camera and an additional external web camera (Logitech HD Pro Webcam C920, Newark, NJ, USA).

In total, seven (*N* = 7) yoga sessions were recorded from April to September 2020. Session 1 was a pilot session, lasting only 50 mins due to software issues; therefore, there are no data from the last part of this practice. There was a problem with the external webcam in sessions 1 and 4, and so this footage is incomplete.

### 3.4. Data Analysis

#### 3.4.1. Video Coding

Physiology data were labeled with names of postures (English translations of Sanskrit) from half primary series in Ashtanga post recording by video coding in BORIS [69]. A total of 57 postures were used in the analysis; see Table A2 in Appendix B for a list of these postures. Information on head position (up, down, side, and uptilted; side and down-tilted; or changing), whether the pose required bilateral, or unilateral muscle activation (left and right sides, respectively), its order in the sequence and which part of the sequence it belonged to (warm-up, standing, seated, or finishing) were added as metadata.

#### 3.4.2. fNIRS Analysis

FNIRS data were prepared in python and analyzed in MATLAB R2020a with NIRS toolbox [65]. The standard pre-processing included conversion from raw data to optical density, then conversion to hemoglobin concentration using the modified Beer–Lambert Law with extinction coefficient from [70], and a partial pathlength factor (PPF) of 0.1. Statistical analysis used a first-level general linear model regression, which used an autoregressive pre-whitening method with iteratively reweighted least-squares (AR-IRLS) [65,71] for motion artifact correction that controls type-I errors. For second-level (group) statistics, a linear mixed-effects model was used, which tested for the main effect of conditions (the yoga postures constitute the conditions). A mixed-effects model was selected since it more effectively accounts for design imbalances and missing values. Then, we ran all permutations of condition contrasts with *t*-tests; i.e., each posture was compared to all other postures. To correct for multiple comparisons, the Benjamini–Hochberg procedure was used, and the corrected *p*-value is denoted as the q-value [72]. The statistical significance level was set at q < 0.05. We refer to [65,71,73] for further details on analysis techniques.

#### 3.4.3. Selection of Contrasts

A number of *P*(*57*,*2*) = 3192 permutations were tested. After removing duplicate contrasts, a total of 1309 contrasts had one or more statistically different channels. We removed postures where the top of the head was facing down or changing position during the posture as this causes increased or unstable blood flow to PFC due to gravity. This returned 939 contrasts. Then, we made sure to only compare postures with the same head position, which resulted in 371 significantly different contrasts. These contrasts were sorted based on the number of significantly different channels. Thereafter, the ten contrasts with the most significantly different channels, the ten contrasts with the least significant different channels, and the ten contrasts with a mid-range number of significantly different channels were selected for visual inspection. The visual inspection of the 30 contrasts determined the postures presented in the results.

## 4. Results

First, we present combinations of postures yielding the greatest number of channels with expected significantly different brain activation or cognitive load. Second, we present posture combinations that show some unexpected differences in activation. Finally, we also highlight some interesting combinations with little significant difference, i.e., postures where we hypothesized differences but were unable to measure any greater significant difference.

### 4.1. Posture Combinations with Expected Greater Statistical Difference

**Boat–Mountain Pose Start.** There was a significant increase in HbO in the Boat compared to the Mountain Pose Start, in 21 channels. Activations occurred along the medial line and parts of r/lPFC, and mDLPFC; see Table 1, row 1, for Hbo visualization, and Table A3 (Appendix C) for statistics. Increased HbO in the PFC, and hemisphere connectivity indicate that the Boat Pose has a higher cognitive load compared to the Mountain Pose. This is an expected finding. Mountain Pose Start is the opening posture of the sequence, by some described as a resting pose. Instructions usually include clearing the head and preparing for the practice. In contrast, Boat is physically demanding in that muscle activation, posture, and balance require presence and concentration. Boat is strenuous, but is claimed to aid in developing concentration stamina, focus, internal awareness, and emotional calmness. Activation of the mFPC may indicate a mental exploration of behavioral changes (e.g., changes in posture and muscle activation) to make the posture easier. The activation predominantly in the mFPC, which is responsible for undirected exploration, may suggest that the practitioner was seeking a new, unknown alternative (as opposed to directed exploration of a known alternative). Activation in the mDLPFC, which plans upcoming behavioral actions, may further suggest that the practitioner had begun to think about and plan upcoming postures. It is possible that there was an attempt to divert thoughts from the strenuous Boat posture.

**Mountain Pose Start–Maricyasana A (MA)** (Marichyasana is a series of four postures (A, B, C, D), each with a difference in form. The original name was kept and a descriptor of the posture added given the many translations from Sanskrit) **Right Knee Bent Bind.** There was a significant decrease in HbO in Mountain pose compared to the MA Right Knee Bent Bind, in 23 channels. Deactivation occurred along the medial line, mFC, and partly the r/lFPC. See Table 1, row 2 for HbO visualization, and Table A4 (Appendix C) for statistics. As expected, Mountain pose is much less cognitively demanding than the MA Right Knee Bent Bind. This seated pose stretches the hamstrings and back by performing a forward fold, and binds the hands behind the back to open the chest. Medial line and m/r/lFPC deactivation suggest mental exploration, perhaps for an improved performance of the posture. Moreover, insignificant DLPFC activity suggests that the practitioner did not divert their attention from this posture to seek alternatives (as opposed to the Boat–Mountain Pose Start contrast above). The MA Right Knee Bent Bind yields a higher cognitive load than Mountain Pose, but it does not seem so intense that the practitioner wants to exit the pose as quickly as in Boat.

**Boat–Lotus.** There was significant increase in HbO in Boat compared to Lotus in 19 channels. Activations occurred in mFPC, some r/lFPC lateralization prevailing in the left hemisphere, and mDLPFC. This indicates a clear difference in cognitive load and state. See Table 1, row 3 for HbO visualization, and Table A5 (Appendix C) for statistics. This result was also expected since Boat was compared to the traditional meditation pose Lotus, which aims to clear the mind. Interestingly, there was less significant activation along the medial line as compared to the Boat–Mountain Pose Start contrast. Activation was predominantly at the montage’s front (mFPC) and back (mDLPFC). This may be due to the greater similarity of these postures, i.e., they are both seated postures, whereas the two former contrasts compare standing and seated postures. We conclude that standing and seated postures have differing requirements.

### 4.2. Postures Combinations with Some Unexpected Differences (Mid-Range)

**Head to Knee Pose B, sit on right heel–Extended Hand to Right Big Toe, hold.** There was a significant increase in HbO in mFPC for Head to Knee Pose B compared to Extended Hand to Right Big Toe Hold. See Table 2, row 1 for HbO visualization, and Table A6 (Appendix C) for statistics. Head to Knee Pose B poses a greater cognitive demand than the Extended Hand to Right Big Toe Hold. This is interesting since we expected the opposite effect. Extended Hand to Right Big Toe imposes a great demand on leg and thigh muscles to statically hold the leg out and up in a straight line while maintaining balance. Additionally, the practitioner was not strong enough to hold their leg straight out and up, but worked hard to not let their foot fall further. Head to Knee Pose B is a seated position where practitioners sit on their heel, folding forward, stretching the hamstring and the back of the leg. The posture is generally considered uncomfortable due to the pressure of the heel on the perineum, especially for novice practitioners. This pain may have caused the unexpected finding. Head to Knee Pose (Sanskrit: Janu Sirsasana) is a hip opener, hamstring stretch, and a slight torso twist, with three variations (A, B, and C) on both the left and right sides, which become incrementally more difficult. A recurring patten of HbO recruitment to mFPC (as depicted in Table 1, row 1) appeared when comparing the Extended Hand to Right Big Toe Hold to both the left and right sides of variation A and B, and the left side of variation C. However, the right side of variation C only recruited D3-S6 and S5-D6, but not S5-D4 (along the medial line), as prior variations did. Since this is the final side in the last variation of this pose, this may suggest the anticipation of and preparation for the next pose, and thus a decreased activation in mFPC overall possibly due to decreasing focus and attention to the posture.

**Lotus Uplifting–Warrior 2 Left.** There was a significant increase in HbO for Lotus Uplifting compared to Warrior 2 Left, lateralized to left hemisphere, in lFPC, lDLPFC. Lotus Uplifting is more cognitively demanding than Warrior 2 Left. See Table 2, row 2 for HbO visualization, and Table A7 (Appendix C) for statistics. In Lotus Uplifting practitioners lift their legs off the ground while maintaining the classical Lotus formation of the legs. It is interesting to see increased activity only in the left hemisphere, suggesting a lateralization of cognitive functions in this contrast. Moreover, when we compared Lotus Uplifting to Warrior 2 Right (same posture performed on the right side), there was no statistical significance in any channels.

**Mountain Pose Start–Extended Hand to Right Big Toe Hold.** There was a significant decrease in HbR in Mountain Pose compared to Extended Hand to Right Big Toe Hold in mDLPFC, and some in lFPC. See Table 2, row 3 for HbR visualization, and Table A8 (Appendix C) for statistics. Decreased HbR in mDLPFC indicates neural activity in this region, which indicates increased cognitive load in Mountain Pose compared to Extended Hand to Right Big Toe Hold. This is an unexpected finding since Extended Hand to Right Big Toe Hold is, as previously described, demanding. Since activity is localized to DLPFC, which activates in anticipation of difficult tasks, it may suggest that the practitioner is mentally preparing themselves for the practice by mentally mapping out coming postures. Therefore, we speculate that there is less need for planning ahead when in Extended Hand to Right Big Toe Hold, due to greater need for concentrating on proper performance during the posture.

### 4.3. Posture Combinations with Unexpected Little Difference

**Lotus Uplifting–Lotus.** There was one significant HbO channel for Lotus Uplifting compared to Lotus in lFPC. Lotus Uplifting is scarcely more cognitively demanding than Lotus. See Table 3, row 1 for HbO visualization, and Table A9 (Appendix C) for statistics. We initially thought that Lotus Uplifting might pose a much higher cognitive demand than Lotus due to the strenuous muscle engagement and coordination required to lift crossed legs from the ground. It is, therefore, interesting that we observed a significant difference in only one channel. We speculate that the participant might have been able to direct their attention to something other than the physical demand, but not so much so that they started planning other action sequences and engaging DLPFC. This may be attributed to their breathing pattern and given cues regarding where to direct their focus; however, we still find this interesting.

**West Side Intense Stretch A and B–Warrior 2 Right.** There was a significant increase in HbO in West Side Intense Stretch A and B compared to Warrior 2 Left, in one channel in m/rFPC. See Table 3, row 2 for HbO visualization, and Table A10 (Appendix C) for statistics. Since Warrior 2 Right is a standing posture requiring balance, hip opening, and a straight back and arms, we initially thought that it would be more demanding than a “simple” seated stretching pose. However, the two variations (A and B) of West Side Intense Stretch had increased HbO in a small region in m/rFPC, and was thus more cognitively demanding than Warrior 2 Left. This may be why its translation from Sanskrit includes the word “intense”.

**Triangle Left–Triangle Right**. There was a slight decrease in HbO in Triangle performed on the left side compared to the right side, in lDLPFC. Triangle Left imposed a slightly lower cognitive load than Triangle Right. See Table 3, row 3 for HbO visualization, and Table A11 (Appendix C) for statistics. A common statement heard in yoga classes is that practitioners may (and probably will) experience side differences. One side may be easier/harder to perform correctly due to differences in flexibility, muscle strength, and anatomy. For this participant, the right side seems more cognitively demanding than the left. This corroborates the participant’s subjective experience of side differences, which is interesting. This difference in activation suggest that subjective experiences of side differences may translate to differences in brain region activation.

## 5. Discussion

### 5.1. Lateralization

An overall observation of contrasts in Table 3 shows that all the lateralization of activation occurs in the left hemisphere. This observation also holds when inspecting Table 1 and Table 2. As found by [51], load-dependent HbO activation yielded stronger activation in the left hemisphere in bilateral DLPFC. This may explain why we observed more activation in the left hemisphere, as it is an indication of increasing cognitive load. Moreover, changes in cognitive state changes functional connectivity in adjacent frontal lobe regions (i.e., FPC and DLPC) measured by HbO [51], which supports the notion that HbO changes found in this study stem from changes in cognitive states.

### 5.2. Reflections on Motion Artifacts

We observed systemic motion artifacts in these data. The practice’s transitions between postures are fast paced. This slightly reduces the optodes pressure on the scalp during the movement across all channels causing spikes in fNIRS data. The practice also includes postures with the head positioned upside-down, e.g., Downward Facing Dog, which causes increased blood flow to the head due to gravity and the head’s position relative to the heart, which adds a baseline shift to the fNIRS data. Postures with the head upside-down can of course not be compared with most of the other postures in which the head is upright. Therefore, we found it helpful to add information on the head position for each posture during the video coding. We did not see any major problems in the fNIRS data for postures with the head positioned on the side, with different tilts or rotations of the head.

As mentioned by [8], head movement, heartbeat, and respiration artifacts may be corrected with filtering, which helps to reduce noise in fNIRS data. The AR-IRLS filter used to process these data is designed for correcting slippage of optodes, motion, and physiological noise by designing optimal pre-whitening filters using autoregressive models and iteratively reweighted least squares [71]. As described in [71], AR-IRLS removes serially correlated errors, and by doing so reduces the false positive rate to 5–9%, which, compared to 37% of ordinary least squares (OLS) with no motion correction, is impressive. We also tested a Temporal Derivative Distribution Repair (TDDR) [74] motion correction method, which allowed us to visually inspect time-series fNIRS data without the visual clutter of artifacts, which was helpful in distilling the insights above (but we did not use it for our analyses here). As outlined by [74], future work on fNIRS motion correction should include whether combinations of two or more correction methods yield improved performance, given the growing number of fNIRS motion correction methods with different strengths and limitations. We look forward to seeing results from these efforts in the coming years.

Despite excellent motion correction methods, for future studies, we obviously recommend keeping gravity where it usually is, but larger changes in, for example, posture and activity performed by participants can be integrated. FNIRS measurement can distinguish between various stimuli within similar contexts, despite noise from the real-world environment and activity.

## 6. Conclusions

This study obtained fNIRS brain activity measurements from seven (*N* = 7) sessions of an Ashtanga Vinyasa Yoga practice conducted in a real-world environment. The results show differences in cognitive load when comparing technically complex postures to relatively simple ones, but also some contrasts with little difference, although a greater difference was initially hypothesized. We now know more about cortical brain activity during a yoga practice. Despite motion artifacts and real-world noise, we can distill cognitive load from applications with considerable motion in the real world, and conclude that it is feasible to obtain neuroimaging measurements in such settings. To the best of our knowledge, this is the first demonstration of fNIRS neuroimaging recorded during any moving yoga practice. It exemplifies that we now have the technologies available for neuroimaging measurements in the real world. This study explores the boundaries of cognitive load measurements in the real world, and contributes to the empirical knowledge base of using fNIRS in realistic settings. Future work with fNIRS should take advantage of this by accomplishing studies with considerable movement in the real world.

## Figures and Tables

**Table 1:** Contrasts postures with greater statistical differences. Contrasts are shown as *t*-tests of Posture 1 minus Posture 2 ^1^.

Posture 1	Contrast Statistics Visualized on Probe Montage	Posture 2
Boat	HbO	Mountain Pose Start
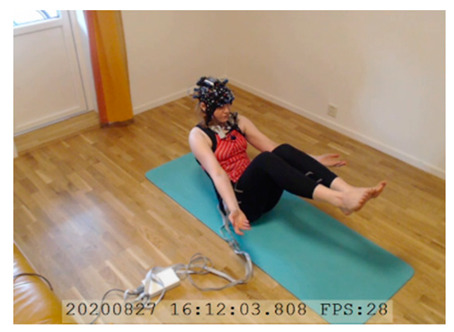	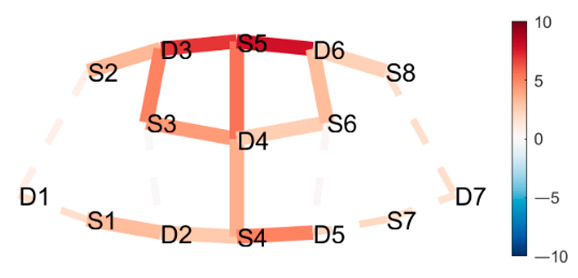	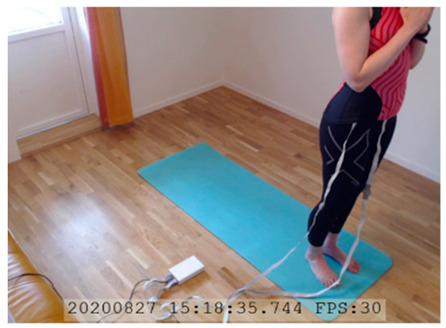
Mountain Pose Start	HbO	MA Right Knee Bent Bind
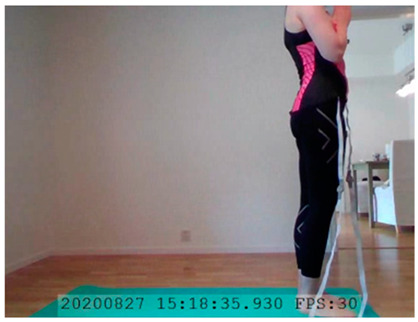	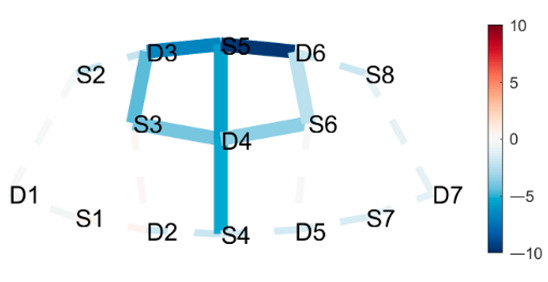	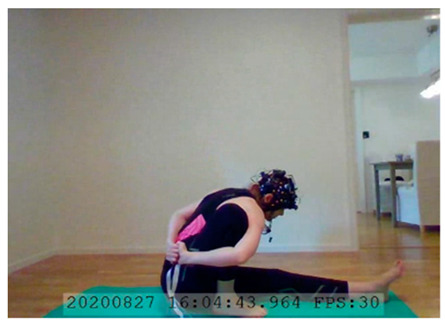
Boat	HbO	Lotus
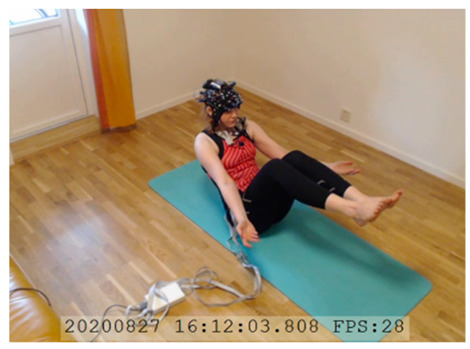	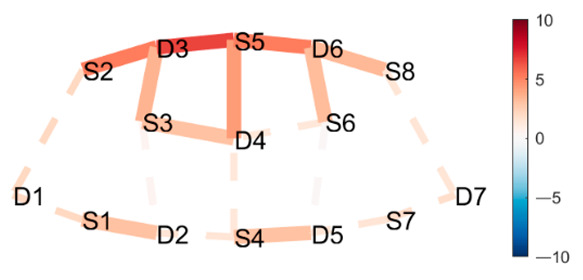	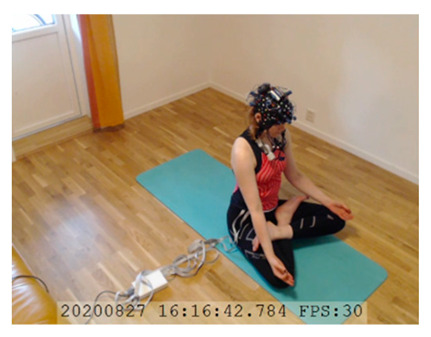

^1^ Second-level analysis of HbO and HbR changes for selected contrasts; t-statistic maps with significantly different brain activation (q < 0.05) in solid lines; color bar represents the t-statistic (scaled to range [−10, 10]) with red/blue indicating statistical increase/decrease in HbO concentration between the postures.

**Table 2:** Contrasts postures with middle significant differences. Contrasts are shown as *t*-tests of Posture 1 minus Posture 2 ^1^.

Posture 1	Contrast Statistics Visualized on Probe Montage	Posture 2
Head to Knee Pose B, sit on right heel.	HbO	Extended Hand to Right Big Toe Hold
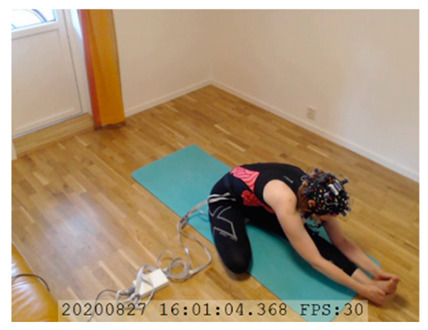	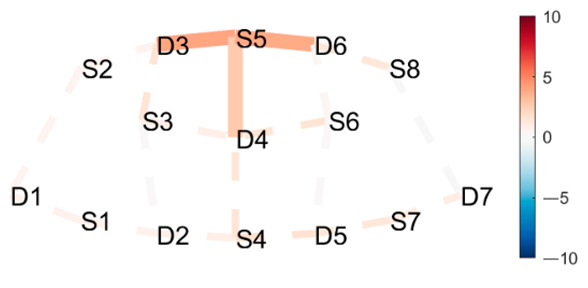	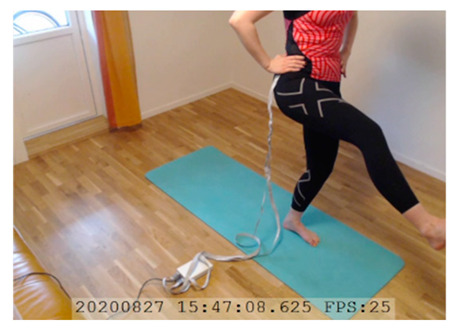
Lotus Uplifting	HbO	Warrior 2 Left
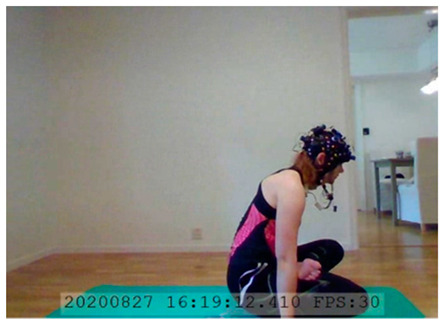	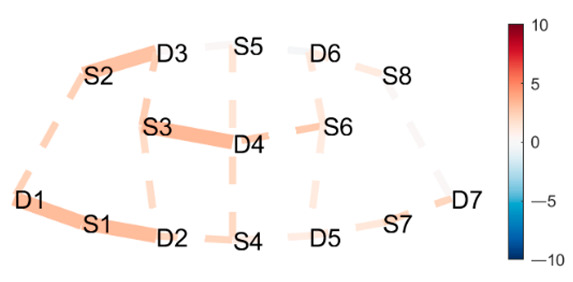	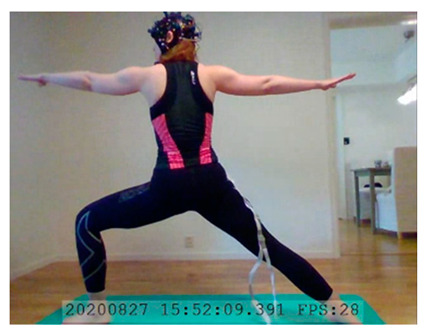
Mountain Pose Start	HbR	Extended Hand to Right Big Toe Hold
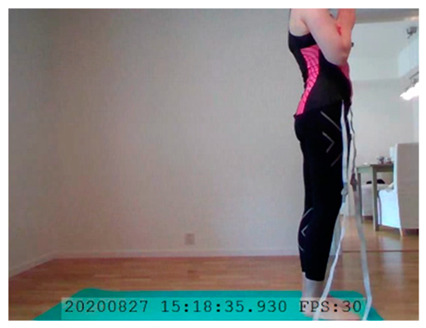	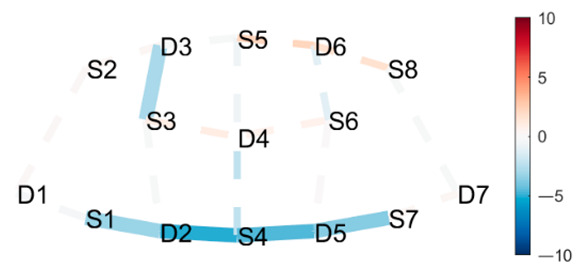	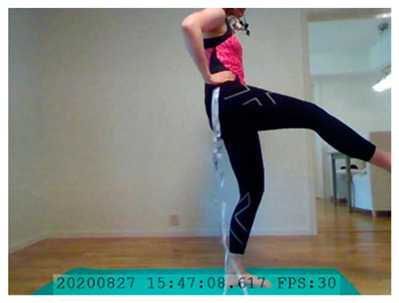

^1^ Second-level analysis of HbO and HbR changes for selected contrasts; t-statistic maps with significantly different brain activation (q < 0.05) in solid lines; color bar represents the t-statistic (scaled to range [−10, 10]) with red/blue indicating statistical increase/decrease in HbO concentration between the postures.

**Table 3:** Contrasts postures with little significant difference. Contrasts are shown as *t*-tests of Posture 1 minus Posture 2 ^1^.

Posture 1	Contrast Statistics Visualized on Probe Montage	Posture 2
Lotus Uplifting	HbO	Lotus
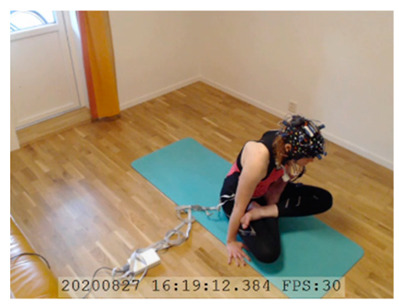	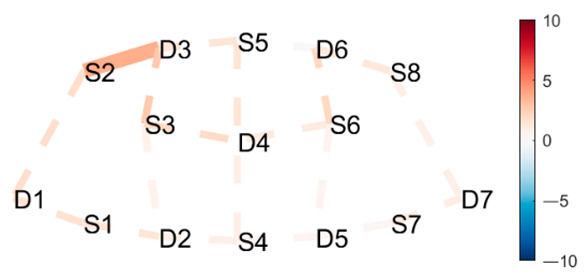	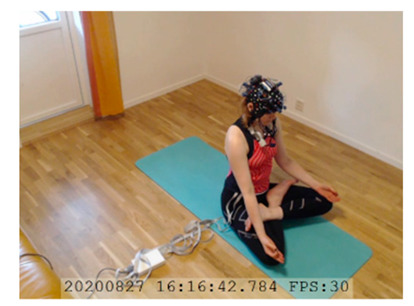
West Side Intense Stretch	HbO	Warrior 2 Right
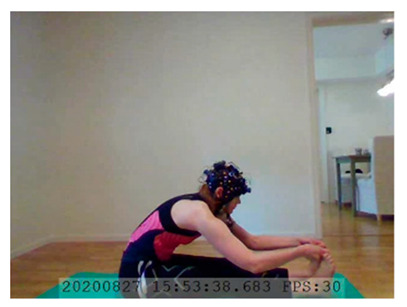	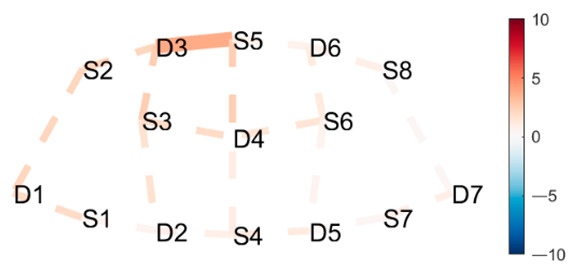	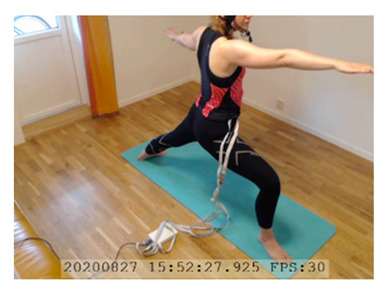
Triangle Left	HbO	Triangle Right
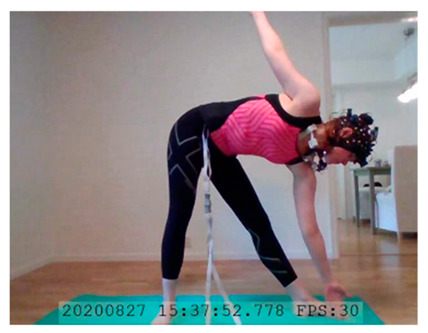	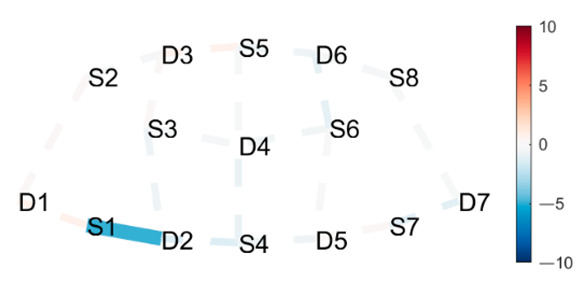	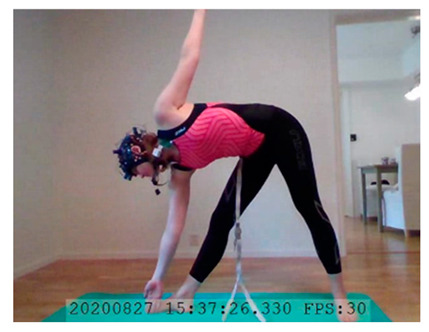

^1^ Second-level analysis of HbO and HbR changes for selected contrasts; t-statistic maps with significantly different brain activation (q < 0.05) in solid lines; color bar represents the t-statistic (scaled to range [−10, 10]) with red/blue indicating statistical increase/decrease in HbO concentration between the postures.

## Data Availability

The data presented in this study are available on request from the corresponding author.

## Appendix A

Table A1 includes the list of the postures that were a part of the practice. Postures were held for 5 breaths unless otherwise stated.

**Table A1:** Practice sheet/list of postures.

Sanskrit	English	Comment
**Warm-up**		
Samasthitih	Mountain Pose	Conducted. Participant listened to chant in video.
Surya Namaskara A x5	Sun Salutation A	Conducted.
Surya Namaskara B x5	Sun Salutation A	Conducted.
**Standing postures**		
Padangusthasana	Big Toe Pose	Conducted.
Pada Hastasana	Hand to Foot Pose/hands under feet	Conducted.
Utthita Trikonasana	Triangle	Conducted.
Parivṛtta Trikonasana	Revolved Triangle	Conducted.
Utthita Parsvakonasana	Extended Side Angle	Conducted.
Parivṛtta Parsvakonasana	Revolved Side Angle	Conducted.
Prasarita Padottanasana, A, B, C and D	Wide Leg Forward Fold A, B, C, and D	Conducted, but adapted to accommodate the sensors. The head was not placed on the ground.
Parsvottanasana	Side Intense Stretch	Conducted.
Utthita Hasta Padangusthasana	Extended Hand to Big Toe Pose	Conducted.
Utthita Parsvasahita	Extended Hand to Big Toe Side and Hold Pose	
Ardha Baddha Padmottanasana	Half Bound Lotus Standing Forward Bend	Conducted.
Utkatasana	Chair Pose	Conducted.
Virabhadrasana I/A	Warrior 1/A	Conducted.
Virabhadrasana II/B	Warrior 2/B	Conducted.
**Seated postures**		
Dandasana	Staff Pose	Conducted.
Pascimottanasana A, B	West Intense Stretch	Conducted.
Purvottanasana	East Intense Stretch	Conducted.
Ardha Baddha Padma Pascimottanasana	Half Bound Lotus Forward Fold	Conducted.
Triyang Mukha Eka Pada Pascimottanasana	One Leg Folded Back, Forward Fold	Conducted.
Janu Sirsasana A, B and C	Head to Knee Pose A, B, and C	Conducted.
Maricyasana A, B C and D	Marichi’s Pose. A seated pose with twist variations.	Conducted.
Navasana x5	Boat	Conducted.
**The first half of the Primary Series is finished**		
Baddha Konasana A and B	Bound Angle Pose A (upright) and B (fold)	Conducted sometimes.
**Finishing sequence**		
Urdhva Dhanurasana	Wheel Pose	Not conducted.
Pascimottanasana—10 breaths	Seated Forward Fold/Bend	Conducted.
Salamba Sarvangasana—10 breaths	Shoulderstand	Not conducted.
Halasana	Plow	Not conducted.
Karna Pidasana	Ear Pressure Pose	Not conducted.
Urdhva Padmasana	Upward Lotus Pose	Not conducted.
Pindasana	Embryo	Not conducted.
Matsyasana	Fish Pose	Not conducted.
Uttana Padasana	Raised Leg Pose	Not conducted.
Sirsasana A and B—10 breaths	Headstand A and B	Not conducted.
Balasana	Child’s Pose	Conducted. The head was not placed on the ground.
Baddha Padmasana (–10 breaths)	Bound Lotus and Bow	Conducted.
Padmasana	Lotus position	Conducted
Utplutih	Scale Pose/Lotus Uplifting	Conducted
Samasthitih	Mountain Pose	Conducted. Participant listened to chant in video.
Savasana	Corpse Pose	Not conducted.

## Appendix B

The labels used for video coding in BORIS can be found in Table A2. These are the postures used in the analysis.

**Table A2:** Video coded postures/list of postures used in the analysis.

Sanskrit	English
Samasthitih (start of practice)	Mountain_Pose_Start
Surya Namaskar A	Sun_Salutation_A
Surya Namaskar B	Sun_Salutation_B
Adho Mukha Svanasana	Downward_Facing_Dog
Padangusthasana	Big_toe_pose
Pada Hastasana	Hands_under_feet
Utthita Trikonasana (right foot forward)	Trikonasana_Right
Utthita Trikonasana (left foot forward)	Trikonasana_Left
Parivrtta Trikonasana (right foot forward)	Revolved_triangle_Right
Parivrtta Trikonasana (left foot forward)	Revolved_triangle_Left
Utthita Parsvakonasana (right foot forward)	Extended_side_angle_Right
Utthita Parsvakonasana (left foot forward)	Extended_side_angle_Left
Parivṛtta Parsvakonasana (right foot forward)	Revolved_side_angle_Right
Parivṛtta Parsvakonasana (left foot forward)	Revolved_side_angle_Left
Prasarita Padottanasana	Wide_leg_forward_fold
Parsvottanasana (right foot forward)	Side_intense_stretch_Right
Parsvottanasana (left foot forward)	Side_intense_stretch_Left
Utthita Hasta Padangusthasana (right toe)	Extended_hand_to_Right_big_toe
Utthita Parsvasahita (right foot uplifted)	Extended_hand_to_Right_big_toe_hold
Utthita Hasta Padangusthasana (left toe)	Extended_hand_to_Left_big_toe
Utthita Parsvasahita (left foot uplifted)	Extended_hand_to_Left_big_toe_hold
Ardha Baddha Padmottanasana (right foot bound)	Right_foot_in_half_bound_lotus
Ardha Baddha Padmottanasana (left foot bound)	Left_foot_in_half_bound_lotus
Utkatasana	Chair_Pose
Virabhadrasana I (right foot forward)	Warrier_1_Right
Virabhadrasana I (left foot forward)	Warrier_1_Left
Virabhadrasana II (right foot forward)	Warrier_2_Left
Virabhadrasana II (left foot forward)	Warrier_2_Right
Dandasana	Staff_pose
Pascimottanasana A, B	West_intense_stretch_A_B
Purvottanasana	East_intense_stretch
Ardha Baddha Padma Pascimottanasana (right foot)	Right_foot_in_half_bound_lotus_forward_fold
Ardha Baddha Padma Pascimottanasana (left foot)	Left_foot_in_half_bound_lotus_forward_fold
Triyang Mukha Eka Pada Pascimottanasana (right leg)	Right_leg_folded_back_forward_fold
Triyang Mukha Eka Pada Pascimottanasana (left leg)	Left_leg_folded_back_forward_fold
Janu Sirsasana A (right leg)	Head_to_knee_pose_A_Right_Leg_folded
Janu Sirsasana A (left leg)	Head_to_knee_pose_A_Left_Leg_folded
Janu Sirsasana B (right heel)	Head_to_knee_pose_B_sit_on_Right_heel
Janu Sirsasana B (left heel)	Head_to_knee_pose_B_sit_on_Left_heel
Janu Sirsasana C (right toe)	Head_to_knee_pose_C_Right_toe_stretch
Janu Sirsasana C (left toe)	Head_to_knee_pose_C_Left_toe_stretch
Maricyasana A (right knee bent up)	MA_Right_knee_bent_bind
Maricyasana A (left knee bent up)	MA_Left_knee_bent_bind
Maricyasana B (left leg Lotus)	MB_Left_leg_lotus_Right_knee_bent_bind
Maricyasana B (right leg Lotus)	MB_Right_leg_lotus_Left_knee_bent_bind
Maricyasana C (right knee bent up)	MC_Right_knee_bent_twist_bind
Maricyasana C (left knee bent up)	MC_Left_knee_bent_twist_bind
Maricyasana D (left leg Lotus)	MD_Left_leg_lotus_Right_knee_bent_twist_bind
Maricyasana D (right leg Lotus)	MD_Right_leg_lotus_Left_knee_bent_twist_bind
Navasana	Boat
Baddha Konasana	Bound_angle_upright_and_fold
Pascimottanasana	Forward_fold_end
Balasana	Childs_pose
Baddha Padmasana	Bound_lotus_bow
Padmasana	Lotus
Utplutih	Lotus_uplifting
Samasthitih (end of practice)	Mountain_Pose_Stop

## Appendix C

**Table A3:** *t*-test statistics for each channel (source–detector pair). Boat–Mountain Pose Start.

Source	Detector	Type	Beta	Se	Tstat	Dfe	Q	Power
1	2	hbo	197.46	60.05	3.29	321	0.00	0.79
1	2	hbr	95.15	20.27	4.69	321	0.00	0.99
2	3	hbo	313.03	90.63	3.45	321	0.00	0.83
3	3	hbo	313.97	60.74	5.17	321	0.00	1.00
3	3	hbr	49.19	13.82	3.56	321	0.00	0.86
3	4	hbo	253.23	58.59	4.32	321	0.00	0.97
4	2	hbo	192.11	71.50	2.69	321	0.02	0.58
4	2	hbr	153.95	21.13	7.29	321	0.00	1.00
4	4	hbo	337.58	92.19	3.66	321	0.00	0.88
4	4	hbr	94.87	30.08	3.15	321	0.00	0.75
4	5	hbo	205.60	40.26	5.11	321	0.00	1.00
4	5	hbr	99.92	14.15	7.06	321	0.00	1.00
5	3	hbo	485.21	68.56	7.08	321	0.00	1.00
5	3	hbr	87.51	24.00	3.65	321	0.00	0.88
5	4	hbo	322.83	57.88	5.58	321	0.00	1.00
5	6	hbo	691.47	87.80	7.88	321	0.00	1.00
5	6	hbr	58.16	19.36	3.00	321	0.01	0.70
6	4	hbo	179.67	71.04	2.53	321	0.02	0.51
6	6	hbo	272.10	85.00	3.20	321	0.00	0.76
7	5	hbr	115.30	21.91	5.26	321	0.00	1.00
8	6	hbo	225.62	94.04	2.40	321	0.03	0.46

**Table A4:** *t*-test statistics for each channel (source–detector pair). Mountain Pose Start–MA Right Knee Bent Bind.

Source	Detector	Type	Beta	Se	Tstat	Dfe	Q	Power
2	3	hbo	−346.99	103.30	−3.36	321	0.00	0.81
2	3	hbr	−109.30	26.27	−4.16	321	0.00	0.95
3	3	hbo	−281.68	75.93	−3.71	321	0.00	0.89
3	3	hbr	−49.84	17.72	−2.81	321	0.01	0.63
3	4	hbo	−215.96	69.01	−3.13	321	0.00	0.74
3	4	hbr	−44.20	15.20	−2.91	321	0.01	0.66
4	4	hbo	−354.04	101.43	−3.49	321	0.00	0.84
4	4	hbr	−107.66	37.51	−2.87	321	0.01	0.65
5	3	hbo	−558.02	80.00	−6.98	321	0.00	1.00
5	3	hbr	−197.23	28.89	−6.83	321	0.00	1.00
5	4	hbo	−346.45	69.69	−4.97	321	0.00	0.99
5	4	hbr	−77.77	20.22	−3.85	321	0.00	0.91
5	6	hbo	−818.60	99.86	−8.20	321	0.00	1.00
5	6	hbr	−101.37	22.93	−4.42	321	0.00	0.97
6	4	hbo	−274.98	82.53	−3.33	321	0.00	0.80
6	4	hbr	−81.21	27.58	−2.94	321	0.01	0.67
6	6	hbo	−351.87	104.43	−3.37	321	0.00	0.81
6	6	hbr	−89.55	25.90	−3.46	321	0.00	0.83
7	5	hbr	−61.91	26.04	−2.38	321	0.03	0.45
8	6	hbo	−462.97	105.39	−4.39	321	0.00	0.97
8	6	hbr	−112.37	32.59	−3.45	321	0.00	0.83

**Table A5:** *t*-test statistics for each channel (source–detector pair). Boat–Lotus.

Source	Detector	Type	Beta	Se	Tstat	Dfe	Q	Power
1	2	hbo	145.86	47.68	3.06	321	0.01	0.71
2	3	hbo	366.15	69.53	5.27	321	0.00	1.00
2	3	hbr	65.47	18.41	3.56	321	0.00	0.86
3	3	hbo	175.83	50.31	3.49	321	0.00	0.84
3	4	hbo	149.83	48.33	3.10	321	0.01	0.73
3	4	hbr	34.79	10.21	3.41	321	0.00	0.82
4	4	hbr	75.27	25.77	2.92	321	0.01	0.67
4	5	hbo	101.00	33.64	3.00	321	0.01	0.69
4	5	hbr	38.79	12.10	3.21	321	0.00	0.76
5	3	hbo	374.76	56.25	6.66	321	0.00	1.00
5	3	hbr	61.96	19.22	3.22	321	0.00	0.77
5	4	hbo	202.77	47.24	4.29	321	0.00	0.96
5	4	hbr	45.67	13.59	3.36	321	0.00	0.81
5	6	hbo	406.25	76.05	5.34	321	0.00	1.00
5	6	hbr	80.07	15.84	5.05	321	0.00	0.99
6	6	hbo	222.71	68.88	3.23	321	0.00	0.77
6	6	hbr	58.18	17.87	3.26	321	0.00	0.78
7	7	hbr	47.33	20.23	2.34	321	0.04	0.44
8	6	hbo	263.54	76.20	3.46	321	0.00	0.83
1	2	hbo	145.86	47.68	3.06	321	0.01	0.71
2	3	hbo	366.15	69.53	5.27	321	0.00	1.00

**Table A6:** *t*-test statistics for each channel (source–detector pair). Head to Knee Pose B, sit on right heel–Extended Hand to Right Big Toe Hold.

Source	Detector	Type	Beta	Se	Tstat	Dfe	Q	Power
5	3	hbo	453.27	111.13	4.08	321	0.00	0.94
5	3	hbr	116.32	36.45	3.19	321	0.00	0.76
5	4	hbo	256.29	91.15	2.81	321	0.01	0.63
5	6	hbo	568.80	145.42	3.91	321	0.00	0.92
5	6	hbr	160.80	31.36	5.13	321	0.00	1.00
5	3	hbo	453.27	111.13	4.08	321	0.00	0.94

**Table A7:** *t*-test statistics for each channel (source–detector pair). Warrier 2 Left–Lotus Uplifting.

Source	Detector	Type	Beta	Se	Tstat	Dfe	Q	Power
1	1	hbo	−715.08	219.45	−3.26	321	0.02	0.78
1	2	hbo	−295.74	92.86	−3.18	321	0.02	0.76
2	3	hbo	−414.64	136.13	−3.05	321	0.03	0.71
3	4	hbo	−292.00	85.87	−3.40	321	0.02	0.82
4	5	hbr	65.32	22.85	2.86	321	0.04	0.64
1	1	hbo	−715.08	219.45	−3.26	321	0.02	0.78

**Table A8:** *t*-test statistics for each channel (source–detector pair). Mountain Pose Start–Extended Hand to Right Big Toe Hold.

Source	Detector	Type	Beta	Se	Tstat	Dfe	Q	Power
1	2	hbr	−86.34	26.26	−3.29	321	0.01	0.79
3	3	hbr	−53.18	18.16	−2.93	321	0.03	0.67
4	2	hbr	−141.34	27.43	−5.15	321	0.00	1.00
4	5	hbr	−88.99	19.47	−4.57	321	0.00	0.98
7	5	hbr	−99.69	27.36	−3.64	321	0.00	0.87
1	2	hbr	−86.34	26.26	−3.29	321	0.01	0.79

**Table A9:** *t*-test statistics for each channel (source–detector pair). Lotus Uplifting–Lotus.

Source	Detector	Type	Beta	Se	Tstat	Dfe	Q	Power
2	3	hbo	359.06	97.59	3.68	321	0.01	0.88

**Table A10:** *t*-test statistics for each channel (source–detector pair). West Intense Stretch A and B–Warrior 2 Right.

Source	Detector	Type	Beta	Se	Tstat	Dfe	Q	Power
5	3	hbo	363.78	95.76	3.80	321	0.01	0.90

**Table A11:** *t*-test statistics for each channel (source–detector pair). Triangle Left–Triangle Right.

Source	Detector	Type	Beta	Se	Tstat	Dfe	Q	Power
1	2	hbo	−393.35	78.26	−5.03	321	0.00	0.99
